# The Danish National Survey of Diet and Physical Activity (DANSDA) 2021–2024: Study Design and Participants Characteristics

**DOI:** 10.3390/nu18091426

**Published:** 2026-04-30

**Authors:** Camilla Christensen, Anja Pia Biltoft-Jensen, Jeppe Matthiessen, Kim Henriksen, Mette Rosenlund Sørensen, Tue Christensen, Ellen Trolle, Sisse Fagt

**Affiliations:** National Food Institute, Technical University of Denmark, 2800 Kongens Lyngby, Denmark; apbj@food.dtu.dk (A.P.B.-J.); jmat@food.dtu.dk (J.M.); kihen@food.dtu.dk (K.H.); mers@food.dtu.dk (M.R.S.); tuchr@food.dtu.dk (T.C.); eltr@food.dtu.dk (E.T.); sisfa@food.dtu.dk (S.F.)

**Keywords:** food intake, public health, nutrient intake, nutritional epidemiology, survey protocol

## Abstract

**Background**: The Danish National Survey of Diet and Physical Activity (DANSDA) is Denmark’s national dietary surveillance system, providing population-level data to support evidence-based government advisory tasks and policymaking, research, and education. **Methods**: DANSDA 2021–2024 is a cross-sectional survey based on a simple random sample of citizens aged 4–80 years from the Danish Civil Registration System. Home visits included structured interviews covering socio-economic status, family composition, ethnicity, lifestyle behaviors and attitudes, health and non-communicable diseases, dietary supplement use, and measurements of anthropometrics and blood pressure. Dietary intake was recorded using a digital or paper-based seven-day food record and a food frequency questionnaire. Physical activity was measured with a pedometer and a seven-day step diary. Participants aged 40–70 years were offered blood sampling for glucose and lipid analyses. **Results**: A total of 4223 individuals participated, with 3824 providing valid food records (97.4% were digital). The response rate was 26.3%. The overall underreporting rate was 24%. The sample was skewed by age, education, income, household type, and region; these variables and sex were used to generate weighting factors. Nearly 1000 blood samples were analyzed for glucose and lipids, with surplus material stored in a biobank. **Conclusions**: DANSDA 2021–2024 provides comprehensive data on diet, physical activity, anthropometry, blood pressure, and blood glucose and lipids. Despite declining response rates and underrepresentation of individuals with lower education and income, weighting procedures support its continued use for national monitoring and research. Strengthening participation and representativeness should be a priority in future survey cycles.

## 1. Introduction

Comprehensive and reliable data on food and nutrition intake serve as a vital evidence base for critical public health functions across Denmark. For this reason, the Danish National Survey of Diet and Physical Activity (DANSDA) has been conducted six times since 1985, with the most recent data collected in 2021–2024. These data have supported the identification of diet- and physical activity–related health challenges, the integration of dietary sustainability considerations, the development of evidence-based public health policymaking (such as culturally adapted dietary guidelines), the evaluation of existing health interventions, and the support of nutrient and chemical risk assessments.

The methodologies employed in the DANSDA surveys conducted between 1995 and 2011–2013 have been previously described [1]. For the DANSDA 2021–2024 survey, these methods were further refined by incorporating technological improvements and expanding the range of measured variables. This resulted in a dataset with detailed dietary intake, pedometer-determined activity, socio-economic status, health-related lifestyle and attitudes, anthropometric measurements, blood pressure, and biochemical risk markers. This article describes the methodological approach, response rate, and key sociodemographic characteristics of participants in the DANSDA 2021–2024 survey.

## 2. Materials and Methods

### 2.1. Survey Design

The Danish National Survey of Diet and Physical Activity 2021–2024 was a nationwide cross-sectional study based on simple random samples from the Danish Civil Registration System (CRS) [2]. The study design was built on the methodological approaches used in previous DANSDA surveys [1]. The current survey consisted of a seven-day food record and step diary using pedometry, a structured face-to-face interview on socio-demographic details, eating practices, health-related lifestyle and attitudes, and anthropometric and blood pressure measurements. Blood samples were collected from adult participants with the main purpose of assessing the prevalence of metabolic syndrome in Denmark and its association with diet and physical activity. Supplementary Table S1 compares methods and outcomes between previous DANSDA surveys and the current survey.

Prior to initiating data collection, a pilot survey with 20 participants was conducted to test procedures related to the home visit, following the official guidelines, including the use of personal protective equipment and other measures intended to minimize the risk of SARS-CoV-2 transmission during the COVID-19 pandemic. The pilot study served primarily as a feasibility study during pandemic conditions; no blood samples were collected, and data were not published.

The survey was conducted under the responsibility of the National Food Institute at the Technical University of Denmark, while the recruitment of participants and data collection were handled by interviewers from a contractor. The DANSDA team and the contracted agency conducted one-day in-person training sessions for interviewers. Instructional videos demonstrating anthropometric measurement techniques were made continuously accessible to interviewers. The National Food Institute provided assessment tools and a structured interview guide to the interviewers. The contractor conducted preliminary data checks, while the National Food Institute performed systematic quality assurance, for example, by searching for outliers and verifying the consistency of reported meals.

The DANSDA 2021–2024 survey received approval from the Research Ethics Committees of the Capital Region, Denmark (H-19033428), and was registered on clinicaltrials.gov (NCT04314882).

### 2.2. Sample Design

The target sample size was approximately 4000 (with valid food records) to ensure sufficient sampling across age and education groups. This sample size is in line with previous DANSDA surveys (Supplementary Table S1) and allows for the inclusion of approximately 1000 children (4–17 years) and more than 200 older adults (71–80 years) in the sample. Because narrower age groups are used for children than for adults (18–70 years), the number of participating children is a crucial determinant of age-specific representativeness and analytical power. The samples were drawn from the CRS each quarter from summer 2021 to spring 2024, and data were collected from September 2021 to July 2024.

**Figure 1:**
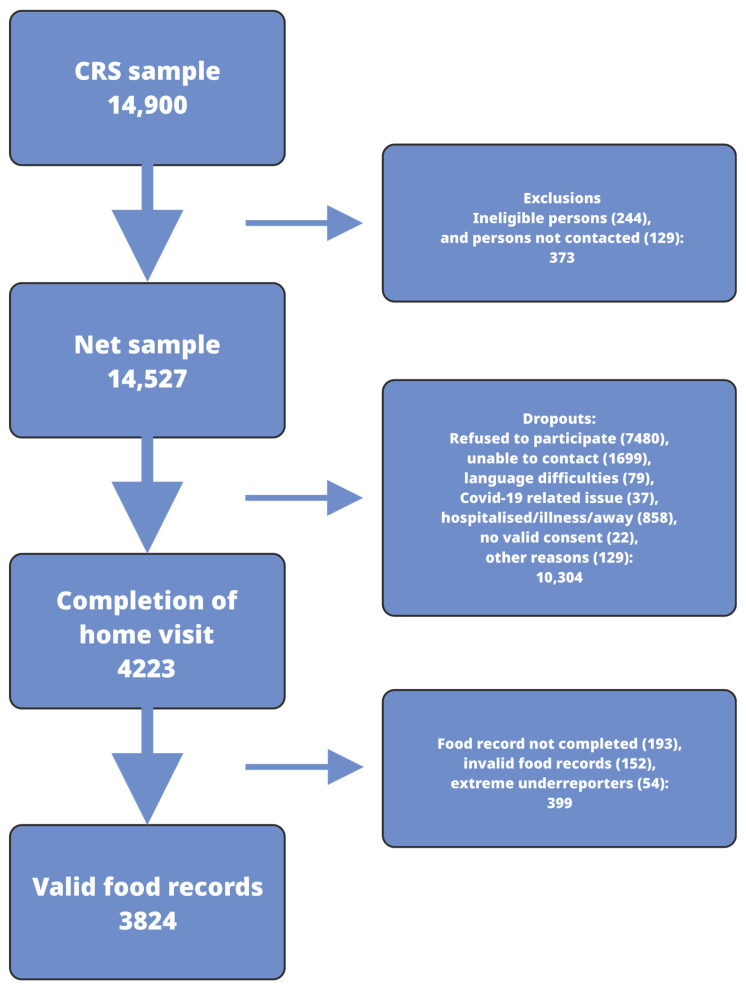
Survey flow chart. CRS: central registration system.

Inclusion criteria were age 4–80 years, sufficient Danish language proficiency, Danish citizenship, and residing in Denmark. Residents of care homes were excluded, as their meals are prepared externally, and therefore, they are often unable to complete food records independently. Individuals who had moved to an unregistered address, emigrated from Denmark, died, been included in a previous sample in this survey, or been institutionalized (e.g., care home) were excluded from the gross sample. Figure 1 shows the participant inclusion flow chart for the survey.

Owing to insufficient staffing at the contracted agency in 2022, 849 individuals from the Q1 and Q2 samples were not initially invited. These individuals were reallocated to Q2 2024, except for those who had reached >80 years of age (*n* = 3). Consequently, no 4- to 5-year-olds were included in the reallocated sample. The reallocation ensured that nearly the entire sample was utilized.

### 2.3. Informed Consent

All participants gave written consent during the home visit after being provided with written and verbal information about the study, including its purpose, procedures, and their rights as participants. A parent or legal guardian of participants under 18 years of age gave written consent on behalf of their child.

### 2.4. Recruitment and Data Collection

Participants received an invitation letter and survey information via secure digital mailbox or postal mail if exempt from digital mail. Within a month, a trained interviewer contacted potential participants by phone. If no phone number was available or phone contact attempts were unsuccessful, the potential participants were contacted in person. Those interested in participating in the survey arranged a time for a visit by the interviewer at the participants’ home address (or another location such as their workplace or a public library). If individuals refused to participate, the reason was recorded if it was stated. Interviewers were instructed to make up to eight phone or text attempts and four in-person visits before classifying participants as dropouts.

During the home visit, participants received further verbal information about the project and were given the opportunity to ask questions. After completing the consent process, instructions for completion of the seven-day food record and step diary were given (including how to use the pedometer). This was followed by the structured interview, and then anthropometric and blood pressure measurements were taken. To enhance response rates, incentives included participation in a cash prize draw and individualized feedback on their results. The participants also kept the Yamax SW200 pedometer.

### 2.5. Measures

#### 2.5.1. Dietary Intake

Dietary intake was estimated using seven-day self-completed digital food records. The food records could be filled out on a computer, tablet, or mobile phone. The seven-day food record has been validated in a previous study [3]. If participants did not wish to use a digital version, paper food records were provided. Participants received instructions from the interviewer for filling in the food records during the home visit. Dietary intake recording was intended to begin a few days after the interview, but it could be postponed if necessary (e.g., due to travel abroad). For 4- to 14-year-old children, parents were responsible for filling in the food record on behalf of their child. Children aged 10–14 years could complete the food record if they were deemed capable by their parents. Parents of 4- to 14-year-old children were also provided with a short version of the paper food record, which could be used when the child was being cared for by others. Parents were asked to transfer the entries in the short paper-based food records to the web-based version.

**Figure 2:**
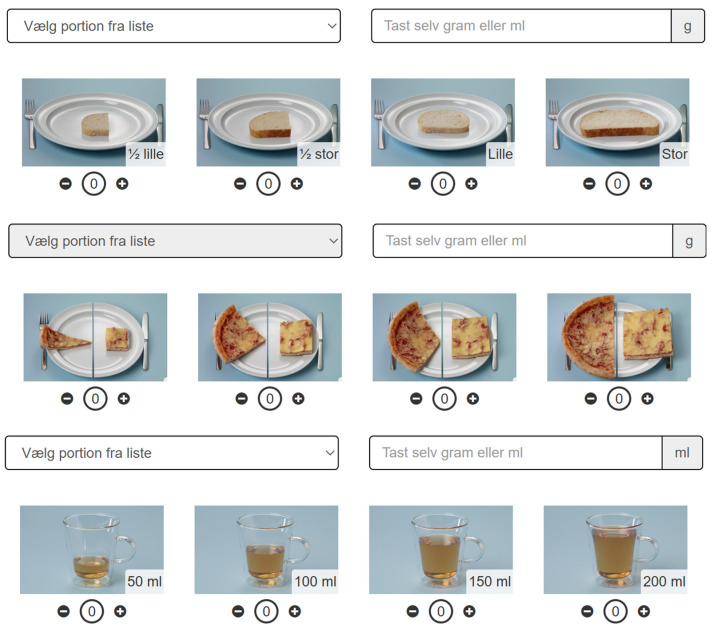
Examples of portion size photos in the digital food record. Top: bread, middle: pizza, bottom: hot beverage. Vælg portion fra liste: choose a portion from the list. Tast selv gram eller ml: enter grams or mL. Lille: small. Stor: large. Participants could either select a fixed portion size from the drop-down list, enter an amount in the box (grams or mL), or indicate the number of portions below the photos using the “−” and “+” buttons.

The digital food record was structured according to the traditional Danish meal pattern, consisting of breakfast, lunch, dinner and three between-meal periods. For seven consecutive days, participants recorded all the foods and beverages they consumed in the food record. Participants recorded their dietary intake by selecting items from a searchable database containing approximately 1700 food and beverage entries. If a specific item was not in the food library, participants could manually enter it as a free-text response. Foods commonly consumed together were suggested as subsequent entries; for example, spread or butter was prompted following the entry of bread. This made it less likely that food would be left out by mistake. Portion sizes were estimated with portion size photos illustrating portion sizes of commonly consumed foods, household measures or in grams or milliliters. Examples of portion size photos are shown in Figure 2. In total, 444 image series illustrating between one and four portion sizes were included.

Dietary supplement use was recorded daily in the food records, including brand and amount consumed (as number of daily doses). Participants selected from ten predefined categories (multivitamin-mineral, iron, vitamin C, strong vitamin D, standard vitamin D, vitamin B_12_, selenium, fish oil, cod liver oil, calcium) or entered open-text responses. The ten predefined categories were chosen based on sales data. A database detailing the nutrient content per daily dose for each supplement was generated to calculate the micronutrient intakes (e.g., mg, µg, α-tocopherol equivalents, etc.) from dietary supplements.

The paper-based seven-day food record mirrored the structure of the digital version, used the same portion size entry options and included a printed photo guide featuring 41 image series, each illustrating six portion sizes. The photo guide has been validated in a previous study [4]. All entries were recorded as free-text responses.

For main meals in both formats of the seven-day food record, participants reported the time the meal started, duration, location, company, and food source.

A seven-day food record qualified as valid if it contained a minimum of three weekdays and one weekend day, as confirmed by the contractor. The National Food Institute then performed a secondary screening to assess completeness and internal validity before inclusion in the analytical dataset.

Incomplete daily records in the dietary registration system were manually checked in-house by the research group. Records still marked as “open” (i.e., not closed by the participant on the final recording day) were reviewed and approved if complete. Dietary data from eligible participants were extracted quarterly from the central database. They were then merged with participant information (age, result codes, etc.) from the home visit database and imported into the Food Intake Coding Tool (FICT) configured for this survey.

Participants also completed a food frequency questionnaire (FFQ) designed to capture the intakes of episodically consumed foods. The FFQ contained questions on energy drinks, sports nutrition products, offal, game meat, seaweed, fish, seafood, flax seeds, rice crackers, nuts, meal replacement products, and vitamin-enriched drinks, as well as plant-based alternatives to dairy, fish and meat. Participants were asked to estimate the average frequency of intake of the food in question in a typical week of the last year. Participants who completed paper-based food records also completed a paper version of the FFQ. The FFQ was developed specifically for this survey based on the dietary assessment guidelines published by the European Food Safety Authority [5]. A separate validation study for the FFQ was not conducted.

Paper-based food records and FFQs were scanned and manually entered in accordance with standardized protocols. Open-ended responses from both digital and paper records were reviewed and coded in FICT. When necessary, new recipes were created in the Web General Intake Estimation System (WebGIES) to accommodate frequently reported foods that could not be coded into existing recipes.

Several quality checks were performed at both the individual and day levels. Days with ≤5 reported items were manually reviewed, and participants with <10 reported items per day on average across the registration period were reviewed for likelihood. Records were examined for missing key components of meals (e.g., main dishes without sides, spreads without bread, breakfast cereals without milk). Extreme intakes were identified using interquartile range (IQR) criteria and investigated on a case-by-case basis.

Invalid days and participants were deactivated in FICT prior to analysis. Energy and nutrient intakes were calculated in WebGIES based on processed and checked records. Information on nutrient content was retrieved from The Danish Food Composition Database, Frida version 5.4 (2025), www.frida.fooddata.dk (accessed on 19 November 2025), provided by the National Food Institute, Technical University of Denmark.

#### 2.5.2. Underreporting of Energy Intake

Energy intake (EI) and basal metabolic rate (BMR) or resting energy expenditure (REE) were calculated for each participant using the method described by Henry (2005) [6] and in the Nordic Nutrition Recommendations 2012 and 2023 [7,8]. An extreme under-reporter was defined as a respondent who had an EI/REE ratio below 0.61 (p2.5). If REE was unavailable for the respondent (*n* = 6), extreme energy reporters were defined as individuals with an energy intake ≥10 × p2.5, corresponding to 6.1 MJ among adults aged 15–80 years.

Under-reporters were identified by comparing reported energy intake (EI) with estimated expenditure using 95% confidence limits and individual REE (EI/REE < 1.1) [9]. The assessment of the prevalence of under-reporting and over-reporting of dietary intakes was performed both at the group level and at the individual level using the Goldberg cut-off method [10], updated by Black (2000) [9], as described by EFSA [5]. A physical activity level (PAL) factor of 1.55 was used for ages 4–10 years and 18–80 years, and for 11- to 17-year-olds, a PAL factor of 1.7 was used. The choice of PAL factors was based on data from the previous DANSDA survey, as pedometer-determined physical activity data for the current survey have not yet been processed and validated [7,11].

#### 2.5.3. Physical Activity

Step-based activity was measured with a Yamax pedometer (Yamax Digi-Walker SW200, Tokyo, Japan), which was checked for counting accuracy by the research team beforehand. The pedometer was worn by participants for seven days during waking hours. Participants were instructed to wear the pedometer on the waistband, in the midline of the right knee. It was only to be removed during water-based activities (e.g., swimming, shower). The step count was recorded in a digital step diary or a paper diary (which was identical to the digital version) at the end of each day. The step diary also included questions about the time of attachment and removal of the pedometer, non-wear time occurring during waking hours, as well as time spent on physical activities not captured by the pedometer, such as cycling and swimming. Furthermore, the participants reported whether they were injured or ill and how and where they fitted the pedometer to their clothes. The step diary was adapted from the Tudor-Locke et al.’s pedometer log [12].

During the interview, the participants were also asked indicator questions about moderate-to-vigorous physical activity (to assess habitual adherence to the physical activity recommendations) and sedentary activities (screen time) during their leisure time. Further details on the definition of valid days of step recording and data processing have been described previously by Matthiessen et al. [13].

#### 2.5.4. Interviews

The structured face-to-face interview was conducted using a CAPI-administered questionnaire. The questionnaire included a wide range of questions on socio-demographic parameters, cooking and eating practices, attitudes toward healthy and sustainable eating, self-assessed health and stress, use of tobacco products, use of food labeling, use of selected medications, dietary supplement use, etc. All questions included predefined response categories, with some offering an additional open-ended response option. The average interview duration was 60 min. Following the interview, photos of dietary supplements were taken to support the accurate interpretation of dietary supplements reported in the seven-day food record. Furthermore, relevant information was collected in relation to blood sample collection when applicable.

#### 2.5.5. Anthropometrics

Anthropometric measurements were taken after the interview and were performed by a trained interviewer using standard equipment in accordance with standard operating procedures. A portable Charder HM200P Portstad (Charder Electronics, Taichung City, Taiwan) (14–210 cm) was used to measure height to the nearest 0.1 cm. All participants were measured twice. If there was a difference of more than 1 cm between the two measurements, a third measurement was performed. Individuals in a wheelchair or bedbound did not have their height measured. Body weight was measured to the nearest 100 g using a Kern MPI set of scales (Kern & Sohn, Balingen, Germany; maximum 200 kg). Body weight was measured twice; if there was more than 0.5 kg between the measurements, a third measurement was performed. Pregnant women, wheelchair users, and individuals with a cast or prosthetic arm or leg were excluded from body weight measurement. Waist circumference was measured twice using a tape measure (SECA 201 (SECA GMBH & Co. KG., Hamburg, Germany, max 205 cm). If there was more than 1 cm between the measurements, a third measurement was performed. Individuals in a wheelchair or with a stoma did not have their waist circumference measured.

#### 2.5.6. Blood Pressure

Blood pressure was measured in 15- to 80-year-old participants after 5 min of seated rest using an A&D UA-1020-W blood pressure monitor (A&D Company, Limited, Tokyo, Japan) with a wide-range cuff suitable for arms ranging from 22 cm to 42 cm in circumference. A&D UA-1020-W is a validated blood pressure monitor [14,15] and meets the requirements of the British Hypertension Society. The monitor automatically measures the blood pressure three times with one minute between each reading. The cuff was placed on a bare arm or thin clothing. The measurements were performed on the left arm as per the SOP with a few exceptions due to physical hindrances (e.g., cast, contraceptive implant, injuries). Participants were instructed not to move or talk during the blood pressure measurement and to avoid consuming food or drinks, as well as using nicotine-containing products or exercising 30 min before the measurements. Pregnancy was an exclusion criterion for blood pressure measurements.

#### 2.5.7. Blood Sampling and Biobanking

Individuals aged 40–70 years were invited to provide a blood sample at one of 38 hospitals or health centers. Participation was voluntary; therefore, only a subset of this age group contributed samples. Blood samples were mailed at ambient temperature to a central hospital for biochemical analysis. All blood samples were analyzed in a central laboratory to ensure consistent methodology and avoid inter-laboratory variation. Surplus samples were stored in a biobank for future research. Fasting glucose, triacylglycerides, HDL cholesterol, and total cholesterol were measured to estimate the prevalence of metabolic syndrome. During the home visit, it was verified that the participants did not meet any of the following exclusion criteria for blood sampling: pregnancy, hemophilia, use of anticoagulants or antiplatelet agents, major surgery in the last two months, type 1 or 2 diabetes, kidney disease, chronic inflammatory gut disease, epilepsy, or cancer.

#### 2.5.8. Statistical Methods

Descriptive statistics are presented for the survey participants and for the general population. Chi-square tests were used to compare proportions. R (version 4) was used for statistical analysis.

## 3. Results

### 3.1. Response Rate

A total of 14,900 individuals were included in the gross sample from the CRS. Due to staff shortages at the contracted agency, 129 individuals were not invited to participate in the survey. When calculating the response rate, we excluded these 129 individuals from the gross sample.

Of the 14,771 individuals, 244 were deemed ineligible and subsequently excluded from the sample. Thus, in total, 14,527 were invited to participate in the survey. Of those invited, 4223 (29.1%) accepted the invitation, provided written consent, and completed a structured interview. Among the 4223 individuals, valid food records were collected from 3824, resulting in a response rate of 26.3%. The drop-out reasons are listed in Figure 1. The primary reason for non-participation was refusal to participate, followed by unsuccessful attempts to contact individuals by phone or at their home address.

### 3.2. Food Records

Of all collected seven-day food records, 95.6% of digital food records and 89.9% of paper-based records were considered valid. Among the 3824 valid records, only 2.6% were submitted on paper. These paper submissions were primarily from participants aged 60–80 years (82%), while 5% were submitted by children.

Interviews and dietary and step data were collected year-round, with reduced activity during major holidays (late December–early January, Easter, and July–early August). Consequently, seasonal variation in dietary intake is captured at the group level.

#### Exclusion of Extreme Under-Reporters and Underreporting of Energy Intake

Fifty-four individuals were classified as extreme under-reporters and were therefore excluded from the dietary intake analyses. Under-reporters who did not meet the criteria for extreme under-reporting remained included in the survey sample. The overall under-reporting rate was 24%, with rates of 8–40% observed among adolescents and adults.

### 3.3. Socio-Demographic Characteristics and Representativeness

To assess the representativeness of the sample, participant characteristics were compared with those of the general Danish population aged 4–80 years (Table 1). There were statistically significant differences in the distributions of age, education, household type, income, and region (*p* < 0.001), but not sex (*p* = 0.08).

Children (<15 years) and adults aged 40–59 and 65–74 years were slightly overrepresented. Adolescents (≥15 years) and adults aged <40, 60–64 and 75–80 years were slightly underrepresented compared to the general population. Educational attainment followed previous DANSDA survey patterns, with a marked underrepresentation of individuals with primary education and an overrepresentation of those with higher education. Equalized disposable family income deviated by about 10%, with higher-income families overrepresented. Regionally, the Capital Region was underrepresented; Zealand and Southern Denmark aligned with the general population. Central and Northern Denmark were slightly overrepresented. Households with two or more adults without children were overrepresented, while other types of households were underrepresented. The “other” category included cases with insufficient household information, especially ≥15-year-olds who did not provide details on whether there was one or two parents in the household.

#### Population Weighting Factors

Although simple random samples were drawn from the Central Register System (CRS), the final survey sample did not fully match the demographic composition of the Danish population. Therefore, in-depth non-response analyses were conducted, and population weighing factors were subsequently generated. Because the survey spanned nearly four years, the non-response analysis was conducted separately for two periods (2021–2022 and 2023–2024) using general population demographic data from 2022 and 2023, respectively, as references. In 2021–2022, the sample was skewed in terms of age, education, and equalized disposable family income. In 2023–2024, the comparison with the general population indicated significant differences in education, age, family type, equalized disposable family income, and geographic region. These five variables, along with sex, were included in the construction of population weights. The resulting weighting factors range from 0.05 to 3.45.

### 3.4. Blood Samples

Of the 1937 participants eligible to provide a blood sample, 80.4% consented during the interview, and 62.2% of those subsequently attended a phlebotomy unit. Supplementary Table S2 shows the characteristics of participants who provided a sample, those who consented but did not, and those who declined. Significant differences were observed between the three groups for sex, age, education, household income, and region, but not urbanization. Among donors, women were overrepresented (57%), as were individuals with a bachelor’s degree and higher disposable income (>300,000 DKK), while those with only primary education and living in the Capital Region were underrepresented compared to those who did not agree to give a blood sample. Comparing the blood sample donors with the general population was not feasible at this stage.

## 4. Discussion

DANSDA 2021–2024 resulted in nationwide data from a random sample of the Danish population on diet, step-based physical activity, anthropometric measures, blood pressure, socio-economic factors, cooking and eating practices, attitudes toward healthy and sustainable eating, and biochemical markers. A total of 4223 individuals participated, with 3824 providing valid food records.

DANSDA’s comprehensive dataset sets it apart from other dietary and nutrition surveys conducted in the Nordic region. In Norway’s 2022/2023 adult diet survey, methods included two 24 h recalls, an online food questionnaire, self-reported anthropometric data, and socio-economic information [17]. Similarly, in Finland, two 24 h recalls and FFQ were used, along with questionnaires collecting data on socio-demographics and physical activity. As the Finnish diet survey in 2017 was part of a national health survey, some anthropometric data were collected at health examinations; otherwise, height and weight were self-reported [18,19]. In Sweden, a four-day digital food record was combined with a 53-item questionnaire on selected foods and background information, and blood and urine samples were collected from a subsample [20]. In the Nordic Monitoring System, food intake, other key health-related outcomes and education were assessed in a short questionnaire [21].

### 4.1. Response Rate

The response rate was lower (26.3%) than in previous DANSDA surveys (e.g., 54% in 2011–2013). However, the response rate was similar to other European surveys performed around the same time [21,22,23]. In a national Danish health survey relying solely on questionnaires, response rates have also declined substantially in recent years (from 56.7% in 2021 to 43.0% in 2025) [24]. In DANSDA, participants received a 1.5 h home visit during which they underwent anthropometric measurements and blood pressure assessments, received instructions for completing the seven-day food records and step diaries, and participated in a structured interview. They then maintained a detailed seven-day food record, wore a pedometer, and recorded daily step counts in a seven-day step diary. In contrast, the Nordic Monitoring System 2024, which was conducted in the five Nordic countries, involved a 15 min interview (phone or web-based questionnaire) on diet, alcohol consumption, physical activity, use of tobacco and nicotine products, body weight, height, and educational level, resulting in minimal participant burden. Yet the response rate was 19% for adults and 20% for children in the Nordic Monitoring System [21]. Additionally, the participant burden in DANSDA 2021–2024 was not notably higher than in DANSDA 2011–2013. Therefore, other factors may have influenced willingness to participate. For instance, increased awareness of the General Data Protection Regulation (GDPR) may have made individuals more cautious about invitations to take part in surveys and provide personal information. In addition, general survey fatigue and concerns about visitors to the home during and after the COVID-19 pandemic may have reduced participation. Finally, many individuals already monitor their diet and physical activity using various apps and may therefore perceive little added value in participating in a diet and physical activity survey.

Although the participant burden was high in DANSDA 2021–2024, the incentives (prize draw and personalized feedback on results, except diet) and in-person home visit may have contributed to participation. Notably, the offer of blood sampling was met with a high acceptance rate, despite the absence of specific incentives to compensate for the added inconvenience of transport to a hospital and fasting prior to sample collection. In the recent Norwegian and Finnish adult diet surveys, the response rates were 37% and 53%, respectively. However, in addition to being less time-consuming for participants, in Norway, all diet survey participants received a monetary incentive and personalized dietary feedback [17]. In Finland, participants also received personalized feedback on dietary intake in addition to the results from the health examination [18].

Reducing the participant burden may increase the response rate in future DANSDA surveys. The seven-day food record and step-based activity remain fundamental components of DANSDA; however, the interview component could be optimized by reducing its length and transitioning to an online format, making participation more convenient and acceptable to more people. Additional incentives, such as personalized dietary feedback, could also be considered. Moving the interview online would, however, require new methods for collecting anthropometric measurements or the use of self-reported weight, height, and waist circumference data.

### 4.2. Dietary Data

Transitioning to a digital food record significantly improved data detail, strengthened data checks, and streamlined data processing. The digital food record incorporated an extensive food and beverage database, along with filters and automated reminders for commonly omitted items such as salt, sweets, chocolate, and beverages. Additionally, the digitalized record may have made it easier to record dietary intakes on the go, as most individuals have a mobile device available throughout the day. Increasing the level of detail in dietary survey data enhances the accuracy of nutrient intake estimations, supports the identification of diet-related health risks, and enables more robust links between diet and long-term health outcomes. While those less comfortable with digital technologies were offered the option of a paper-based food record, very few chose to use it. Nonetheless, the overall rate of underreporting of energy intake was 24%, which was comparable to the estimated rates in the three previous DANSDA surveys [1].

### 4.3. Physical Activity

Step counts have been consistently measured with the same Yamax pedometer since the DANSDA 2011–2013 survey, making DANSDA one of the few national surveys providing objective, longitudinal assessments of step-based activity [25]. Transitioning to a digital step diary in 2021–2024 replaced most open-ended responses with closed ones, removed some items (e.g., work/school time), added new questions (e.g., use of electric bicycle), refined existing items (e.g., wear compliance), and enhanced data quality via control filters. Monitoring ambulatory physical activity trends over time is essential, especially as the growing use of e-scooters and electric bicycles may influence activity levels depending on whether these modes replace walking, regular cycling, or more passive transport such as cars or public transport [25,26].

### 4.4. Blood Sampling and Biobanking

Nearly 1000 blood samples were successfully collected, meeting the study group’s target. As blood sampling was voluntary, only a subset of the 40- to 70-year-old participants provided samples, which may introduce selection bias into subsequent analyses. Future analyses will compare those providing blood samples with the general population. However, preliminary observations indicate that women were overrepresented among donors compared to both non-donors and the general population. Differences in factors such as education, household income, and region between donors, non-donors, and decliners cannot be excluded, indicating that the use of weighting factors will likely be important in future data analyses. Exclusion criteria may also have led to the omission of individuals at higher risk for metabolic syndrome, potentially underestimating its prevalence. The blood samples will support future analyses of associations between diet, physical activity, and health, as well as validation of selected biomarkers. Stored biobank material may also enable additional nutritional and health-related analyses in the future.

### 4.5. Strengths and Limitations

A major strength of DANSDA 2021–2024 is its comprehensive and standardized data collection combining detailed seven-day dietary records with objectively measured step-based activity, anthropometry, blood pressure, and blood-based biomarkers within a national sample. The long-standing survey design and largely comparable methodology across survey cycles allow monitoring of time trends in diet, physical activity, and related health indicators. In addition, the collection and storage of blood samples strengthen the dataset by enabling analyses of relationships among diet, physical activity, and health, as well as biomarker validation, while the biobank will support future nutritional and health assessments.

Major limitations of DANSDA, as in all dietary surveys, include response rate and underreporting. In the present survey, the response rate was lower than in previous DANSDA surveys, and the survey population was not fully representative of the Danish population with respect to education, age, income, household type, and region. Declining response rates and differential non-response may lead to selection bias if participation is associated with health behaviors, socio-economic status, or health consciousness, potentially resulting in more favorable lifestyle profiles among participants than in the general population. Although weighting procedures will be applied in subsequent analyses to improve representativeness, residual bias arising from non-response and unmeasured or behavior-related selection mechanisms may persist [27,28]. Underreporting may affect the estimates of nutrient intakes and thus influence associations between dietary outcomes and other measured factors (e.g., disease, blood pressure, biomarkers). In addition, dietary misreporting may affect the evidence base underpinning dietary guidelines and public health policies [29,30,31].

Furthermore, blood samples were collected from a subsample of participants aged 40–70 years, which may have introduced additional selection bias.

Although not a limitation per se, it may also be worth considering that the long data-collection period may have introduced temporal heterogeneity in exposures and dietary behaviors, especially given changes in pandemic-related conditions from 2021 to 2024. Evidence from a survey conducted during the first national lockdown in Denmark in 2020 showed that both dietary habits and physical activity were affected [32]. Although data were not collected during Danish national lockdowns in DANSDA 2021–2024, residual or evolving pandemic-related effects on behaviors cannot be fully excluded.

## 5. Conclusions

DANSDA 2021–2024 is a nationwide survey of dietary habits and physical activity in the Danish population, providing a unique and comprehensive dataset on 4223 individuals. The collection of blood samples further expanded the research potential of DANSDA 2021–2024 compared with previous DANSDA surveys, as it enables the measurement of health-related biomarkers and biomarkers of nutrient intake. The introduction of digital food record and step diary has streamlined data collection and improved quality. However, response rates have continued to decline compared to previous DANSDA surveys, and the sample is skewed by education, age, income, household type, and region. To address this, population weights have been generated to improve the representativeness of the Danish population. Methodological development will continue with the aim of reducing non-response and improving recruitment of underrepresented groups. These data remain a cornerstone for government advisory tasks, policy development, and research and teaching in diet and health-related fields.

## Figures and Tables

**Table 1:** Comparison of the DANSDA population with valid food records (*n* = 3824) and the general population aged 4–80 years.

	DANSDA 2021–2024	General Population 2023 ^a^
**Sex, *n* (%)**		
Men	1861 (48.7)	2,417,948 (50.1)
Women	1963 (51.3)	2,410,081 (49.9)
**Age (years), *n* (%) ^b^**		
4–9	305 (8.0)	331,518 (6.9)
10–14	317 (8.3)	293,259 (6.1)
15–19	214 (5.6)	321,740 (6.7)
20–24	183 (4.8)	323,560 (6.7)
25–29	201 (5.3)	337,684 (7.0)
30–34	216 (5.6)	321,590 (6.7)
35–39	203 (5.3)	277,533 (5.7)
40–44	231 (6.0)	278,218 (5.8)
45–49	303 (7.9)	330,996 (6.9)
50–54	292 (7.6)	353,757 (7.3)
55–59	338 (8.8)	389,073 (8.1)
60–64	261 (6.8)	344,881 (7.1)
65–69	299 (7.8)	311,695 (6.5)
70–74	235 (6.1)	286,965 (5.9)
75–80	226 (5.9)	325,560 (6.7)
**Education, *n* (%) ^c^**		
Primary or still in school	375 (9.8)	1,077,389 (22.3)
Upper secondary	266 (7.0)	432,301 (9.0)
Vocational	1163 (30.4)	1,463,049 (30.3)
Short-cycle tertiary	308 (8.1)	247,526 (5.1)
Bachelor’s or equivalent	965 (25.2)	842,636 (17.5)
Master’s or equivalent or higher	713 (18.7)	765,128 (15.3)
Missing information	34 (0.9)	N/A
**Household type, *n* (%)**		
Multiple adults, without child(ren)	1406 (36.8)	1,223,705 (28.7)
Multiple adults, with child(ren)	1336 (34.9)	401,854 (37.6)
Single adult, no child(ren)	688 (18.0)	1,386,816 (25.3)
Single adult, with child(ren)	194 (5.1)	1,815,654 (8.3)
Other	200 (5.2)	N/A
**Equivalized disposable family**		
**income (DKr/year), *n* (%) ^d^**		
<300,000 (and unknown)	1.599 (41.8)	2,482,089 (51.4)
>300,000	2.225 (58.2)	2,345,940 (48.6)
**Region, *n* (%)**		
Capital	1053 (27.5)	497,773 (30.6)
Zealand	532 (13.9)	1,127,871 (14.7)
Southern Denmark	758 (19.8)	1,011,836 (21.0)
Central Denmark	1013 (26.5)	1,478,514 (23.4)
Northern Denmark	468 (12.2)	712,035 (10.3)

^a^ Data sourced from Statistics Denmark. Data as of 1 October 2023, with the exception of education (30th September) and annual equalized disposable family income in 2023. The general population included Danish citizens currently residing in Denmark. ^b^ Age of DANSDA participants at the time of the interview and registration of diet and step-based activity. Two participants turned 81 before the interview took place, and they are included in the 75- to 80-year age group. ^c^ Educational attainment data is the highest level of completed education. For children aged 4–14 years, the parents’ education was recorded. Educational level was classified according to a Danish version of the International Standard Classification of Education (ISCED): DISCED-15 [16]. ^d^ Disposable family income is equivalized to account for household size.

## Data Availability

In accordance with Danish law and GDPR regulations, the data and analytical scripts used in this survey are stored on secure servers at the Technical University of Denmark. Access to these materials requires a Disclosure Declaration and can be granted upon request to applicants who meet the eligibility criteria. Requests for access and Disclosure Declarations may be directed to the Technical University of Denmark via email at: dansda@food.dtu.dk. Once fully available, the data will be made available to EFSA and will be accessible in EFSA’s Comprehensive Food Consumption Database.

## Supplementary Materials

The following supporting information can be downloaded at https://www.mdpi.com/article/10.3390/nu18091426/s1, Table S1: Description of the DANSDA surveys from 1995 to 2021–2024 according to sampling method, population sample, and data collection. Table S2: Characteristics of blood sample donors and non-donors.

## Abbreviations

The following abbreviations are used in this manuscript:

DANSDA	Danish National Survey of Diet and Physical Activity
CRS	Central Register System
FFQ	Food Frequency Questionnaire
FICT	Food Intake Coding Tool
CAPI	Computer-Assisted Personal Interviewing
PAPI	Paper-and-Pen Interviewing
DPAQ	Danish Physical Activity Questionnaire
NPAQ	Nordic Physical Activity Questionnaire
GIES	General Intake Estimation System
EI	Energy Intake
BMR	Basal Metabolic Rate
DISCED	Danish version of the International Standard Classification of Education
PAL	Physical Activity Level
IQR	Interquartile Range
REE	Resting Energy Expenditure
