# Supplementary material for: The Danish National Survey of Diet and Physical Activity (DANSDA) 2021–2024: Study Design and Participants Characteristics
Source: Nutrients. 2026 Apr 30;18(9):1426. doi: 10.3390/nu18091426 (PMC13164638; doi:10.3390/nu18091426)
Supplement: Supplementary file 1 [file nutrients-18-01426-s001.zip › nutrients-4240805-supplementary.pdf]

Supplementary Materials

Supplementary Results

Supplementary Table S1. Description of the DANSDA surveys from 1995 to 2021-2024 according to sampling method, population sample, and data collection. Adapted from [1].

DANSDA	1995	2000-2002	2003-2008	2011-2013	2021-2024
Sampling method	Stratified sample	Simple random sample	Simple random sample	Simple random sample	Simple random sample
Age	1-80 years	4-75 years	4-75 years	4-75 years	4-80 years
Total sample size, (Valid participants)	4,771 (3,098)	8,200 (4,120)	8,360 (4,431)	7,358 (3,946)	14,527 (3,824)
Contractor	Private company	Public company	Public company	Public company	Private company
**Data collection**					
Data collection period	January to February 1995, April to May 1995, and August to September 1995	June 2000 to December 2002	May 2003 to May 2008	August 2011 to September 2013	September 2021 to July 2024
Dietary recording	7-day food diary (Paper)	7-day food diary (Paper)	7-day food diary (Paper)	7-day food diary (Paper)	7-day food diary (Web and paper)
Portion size Photographs	5	12	12	41	444
Number of foods	170	562	652	463	1,708
Physical activity (PA)	Indicator questions in the interview	7-day physical activity diary (Paper), DPAQ	7-day physical activity diary (Paper), DPAQ	7-day step-diary (Paper) NPAQ, Pedometer	7-day step-diary (Web and paper) NPAQ, Pedometer
Face-to-face interviews	PAPI	PAPI	CAPI	CAPI	CAPI
Number of survey questions	79	88	84	138	140
Anthropometric measures	Self-reported	Self-reported	Self-reported	Self-reported and device-based measured	Objective measures
Blood pressure	-	-	-	-	≥ 15 years
Blood sampling	-	-	-	-	40-70 years

Initiatives to increase participation rate	- Small gift	- Monetary incentives	- Monetary incentives<br>- Collected food/PA diary from participants home	- Monetary incentives<br>- Keep pedometer<br>- Collected food/PA diary from participants home	- Monetary incentives (prize draw)<br>- Keep pedometer<br>- Food/PA diary digitalized<br>- Feedback

DPAQ: Danish Physical Activity Questionnaire; NPAQ: Nordic Physical Activity Questionnaire; PAPI: Paper-And-Pen interviewing; CAPI: Computer-Assisted Personal Interviewing

1. Graff, H.; Biltoft-Jensen, A.P.; Matthiessen, J.; Fagt, S. The Danish National Survey of Diet and Physical Activity (DANSDA) 1995-2011-2013: Study Design, Study Participants, Participation Rate and Underreporting. *Scand. J. Public Health* **2024**, *52*, 752–760. https://doi.org/10.1177/14034948231190681.

Supplementary Table S2. Characteristics of blood sample donors and non-donors.

	Blood sample donors	Agreed but did not donate	Declined to give a blood sample	P-value[c]
**Sex, *n* (%)**				
Women	547 (57)	272 (46)	183 (48)	<0.001
Men	421 (43)	317 (54)	197 (52)	
**Age (years), *n* (%)**[a]				
40-49	272 (28)	206 (35)	97 (26)	0.003
50-59	358 (37)	205 (35)	130 (34)	
60-70	338 (35)	178 (30)	153 (40)	
**Education, *n* (%)**[b]				
Primary or still in school	47 (4.9)	52 (8.8)	40 (11)	<0.001
Upper secondary	21 (2.2)	12 (2.0)	10 (2.6)	
Vocational	365 (38)	244 (41)	148 (39)	
Short-cycle tertiary	93 (9.6)	50 (8.5)	37 (9.7)	
Bachelor's or equivalent	284 (29)	136 (23)	74 (19)	
Master's or equivalent or higher	158 (16)	95 (16)	70 (18)	
Missing information	0 (0)	0 (0)	1 (0.3)	
**Household income (DKr/year), *n* (%)**				
<300,000	57 (5.9)	67 (11)	40 (11)	<0.001
300,000-599,999	240 (25)	140 (24)	101 (27)	
600,000-899,999	323 (33)	153 (26)	106 (28)	
≥900,000	298 (31)	183 (31)	92 (24)	
Don't know/not willing to answer	50 (5.2)	46 (7.8)	41 (11)	
**Region, *n* (%)**				
Capital	236 (24)	176 (30)	132 (35)	<0.001
Zealand	163 (17)	84 (14)	44 (12)	
Southern Denmark	225 (23)	103 (17)	61 (16)	
Central Denmark	222 (23)	141 (24)	102 (27)	
Northern Denmark	122 (13)	85 (14)	41 (11)	

[a] Age at the time of the interview and registration of diet and step-based activity. [b] Educational attainment data is the highest level of completed education. Education was classified according to a modified version of the International Standard Classification of Education (ISCED-11) [17]. [c] Chi square test.
